# Symptoms indicative of early knee osteoarthritis after ACL reconstruction: descriptive analysis of the SHIELD cohort

**DOI:** 10.1016/j.ocarto.2025.100576

**Published:** 2025-01-30

**Authors:** Anna Cronström, May Arna Risberg, Martin Englund, Dorthe B. Strauss, Paul Neuman, Carl Johan Tiderius, Eva Ageberg

**Affiliations:** a Department of Health Sciences, Faculty of Medicine, Lund University, Lund, Sweden; b Division of Orthopedic Surgery, Oslo University Hospital, Oslo, Norway; c Department of Sports Medicine, Norwegian School Sport Sciences, Oslo, Norway; d Department of Clinical Sciences Lund, Orthopaedics, Faculty of Medicine, Lund University, Lund, Sweden; e Norwegian Sports Medicine Clinic (Volvat NIMI), Oslo, Norway; f Department of Clinical Sciences Malmö, Faculty of Medicine, Lund University, Sweden

**Keywords:** Knee injury, Classification criteria, Patient characteristics

## Abstract

**Objective:**

To describe the SHIELD cohort in terms of symptoms indicative of early knee osteoarthritis (OA) and to investigate associations between patient characteristics (demographics, activity/injury-related) and these symptoms at 1 (cross-sectional) and 3 years (longitudinal) post anterior cruciate ligament reconstruction (ACLR).

**Method:**

106 participants (50 ​% women, mean [SD] age 25 [5] years) were included. Symptoms indicative of early knee OA were evaluated by the Knee injury and Osteoarthritis Outcome Score (KOOS) subscale pain, KOOS subscale pain ≤72 (KOOSpain ≤72), and ≤85 on two out of four KOOS subscales (pain, symptoms, activity of daily living, quality of life) (modified Luyten).

**Results:**

Mean (SD) KOOS pain scores were 83.2 (15.7) and 87.3 (12.7) at 1 and 3 years, respectively. At 1 year and 3 years post ACLR, 18/101 (18 ​%) and 14/86 (16 ​%) participants met the KOOSpain ≤72 criterion, whereas 83/101 (82 ​%) and 67/86 (78 ​%) met the modified Luyten criterion. 7/15 (47 ​%) (KOOSpain ≤72) and 59/70 (84 ​%) (modified Luyten) classified as having knee OA symptoms 1 year post ACLR were still classified as having OA symptoms after 3 years. Lower activity level at 1 year was the sole variable consistently associated with all three outcomes 3 years post ACLR.

**Conclusion:**

The proportion of participants fulfilling existing classification criteria for symptoms indicative of early OA after ACLR is highly dependent on the criteria applied and different criteria seem to capture varying aspects of early OA symptoms. Future studies will reveal if these symptoms will persist long-term or just reflect more transient issues.

## 1 Introduction

A history of anterior cruciate ligament (ACL) injury is reported to be associated with a four to six-fold increased risk of developing knee osteoarthritis (OA) [1], with similar rates among surgically and non-surgically treated individuals [2]. Early detection of knee OA is important as this may permit early intervention before the structural derangement becomes too severe. There is, however, no consensus on the definition of early knee OA [3] and several approaches including different combinations of radiographic, demographic, and symptomatic measures as well as clinical examinations, have been used [4]. Given the slow development of radiographic features, such as osteophytes, radiography may not be a justifiable measure for detecting early knee OA [[5], [6], [7]].

Patient-reported pain and symptoms may be more valid markers of early knee OA since studies report an association between these measures and future severe pain, cartilage thickness, joint space narrowing and knee OA symptoms [[8], [9], [10], [11]]. In addition, post-operative pain after ACLR seems to be important for the prediction of future total knee arthroplasty in patients with ACL-injury [11]. A few different criteria for symptoms indicative of early knee OA have been proposed. In 2003, Englund et al., (Englund criteria) introduced the following definition, using the Knee injury and Osteoarthritis Outcomes Score (KOOS), to define a “symptomatic knee”; KOOS quality of Life (QoL) ≤87.5 and at least 2 of the 4 remining KOOS domains to be: pain ≤86.1, symptoms ≤85.7, activity of daily living ≤86.8, or sport/recreation ≤85.0. This was considered to represent knee issues severe enough for a patient to seek medical care [12]. In 2015, Wasserstein et al. used the Englund criteria as well as a score of ≤72 on the Knee injury and Osteoarthritis Outcomes Score (KOOS) pain subscale as representative of knee OA symptoms and a reduction of 10 points on the KOOS pain subscale as representative of a clinically significant worsening in knee pain up to six years after ACL-reconstruction (ACLR) [13]. In 2018, Luyten et al. proposed that a score of ≤85 on at least 2 out of 4 KOOS subscales (pain, symptoms, activity of daily living and QoL together with clinical signs, such as joint line tenderness or crepitus, and a Kellgren & Lawrence grade of ≤1 may represent early knee OA [14]. Cross-sectional studies applied a modification to the original criteria introduced by Luyten et al., using only the KOOS cut-off (i.e., excluding clinical signs and the Kellgren & Lawrence grade) as representative of knee OA symptoms 6 months [15] and 5 years [16] post ACL injury. There seems, however, to be a discrepancy in the classification of patients with early knee OA symptoms after ACLR depending on the criterion that is used [13,15]. For example, in the study by Wasserstein et al. [13], 9 ​% met the criterion of a score of ≤72 on the KOOS pain subscale, whereas 39 ​% met the Englund criteria for symptomatic knee 2 years after ACLR [12]. In the study by Harkey et al. [15], 24 ​% met the Englund criteria whereas 36 ​% met the modified Luyten criteria 6 months post ACLR. Also, in this population, few studies have investigated whether early knee OA classified at one time point will persist long-term, or if these symptoms will improve over time and are therefore no longer classified as early knee OA. Although Wasserstein et al. classified 9 ​% of the cohort as having early knee OA at both 2 years and 6 years post ACLR using the cut-off ≤72 on the KOOS pain subscale [13], it is not clear if these proportions represented the same participants at the two time points. Using the modified Luyten criteria, another study concluded that approximately 22 ​% of their cohort had persistent OA symptoms from 6 to 12 months post ACLR [17]. It is commonly recommended that the rehabilitation duration for improving symptoms, restoring function and return to pre-injury activity after ACLR should be 9–12 months [18]. Investigating the persistence of early OA symptoms beyond 12 months may, thus, give increased understanding regarding optimal timing for interventions targeting OA symptoms in this population.

Factors contributing to early knee OA symptom development after knee injury are still not fully established, but joint load [[19], [20], [21], [22]] and lower extremity muscle function, such as knee extension strength, have been suggested to play an important role, although the evidence is scarce [16,23]. We are, therefore, in a prospective cohort study (SHIELD) investigating associations between muscle function and features indicative of early knee OA one and three years after ACLR [24]. The aims of the present descriptive study were to describe the SHIELD cohort with regards to 1) patient characteristics and 2) symptoms indicative of early knee OA at 1 year and 3 years post ACLR, and 3) explore any associations between patient-characteristics at 1-year and symptoms indicative of early knee OA at 1 year and 3 years post ACLR, respectively.

## 2 Methods

The SHIELD prospective cohort study [24] is registered at ClinicalTrials.gov (NCT03473873). This is a descriptive study of the SHIELD cohort and adheres to the Strengthening The Reporting of Observational studies in Epidemiology guidelines [25].

### 2.1 Participants

**Fig. 1:**
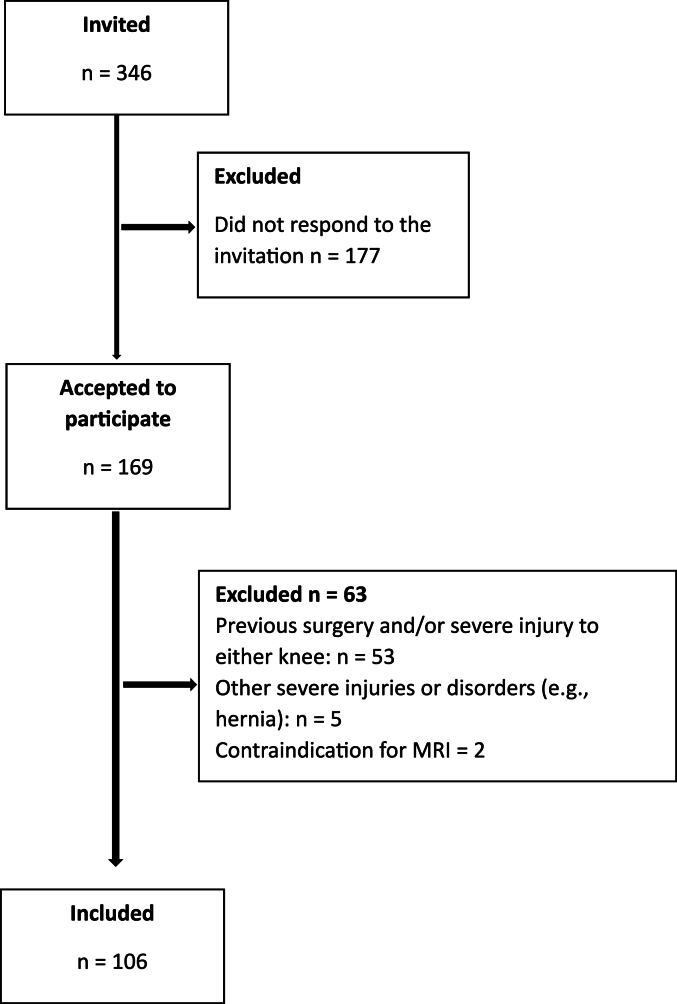
Flow chart of the inclusion process.

Approximately one year (mean (SD) 12 (1.5) months) post ACLR, 106 participants (50 ​% females, mean [SD] age 25 [5] years were included. Participants were recruited from the Department of Orthopedics, Skåne University Hospital, Sweden (n ​= ​75) or the Division of Orthopedic Surgery, Oslo University Hospital, Oslo, Norway (n ​= ​31) between 2018 and 2021. *Inclusion criteria*: i) 10 months to 16 months post ACLR, with or without associated injuries to other structures of the knee, ii) age 18–35 years. *Exclusion criteria*: i) previous serious injury or surgery to either knee, ii) diseases or disorders overriding the knee condition (e.g., neurological disease), iii) contra-indicators for MRI, iv) not understanding a Scandinavian language or English). Full description of the original study is published in a study protocol [24]. The study was approved by the Swedish Ethical Review Board, Sweden (Dnr 2016/319) and the Norwegian Ethical Review Board, Norway (REK sør-øst D: 2016/1128) (See Fig. 1 for flow chart of the inclusion process).

### 2.2 Data collection

#### 2.2.1 Participant characteristics

The following data was collected at 1 year post ACLR (baseline); Sex, age, body mass index, family history (siblings/parents/grandparents) of OA (dichotomous reply [yes/no]), time between injury and surgery (months), graft type, injury mechanism (contact/non-contact), associated injuries (meniscal, cartilage, medial collateral ligament, lateral collateral ligament), activity level (pre-injury and current Tegner activity scale 1 (lowest) – 10 (highest)) [26] and return-to-sport (dichotomous reply [yes/no]). In addition, data on any serious re-injury to either knee were collected at follow-up.

#### 2.2.2 Symptoms indicative of early knee OA

At 1 year and 3 years post ACLR, we used the KOOS subscales pain, symptoms, ADL and QoL, with scores ranging from 0 (worst) to 100 (best) [27]. Based on the proposed different criteria of symptoms indicative of early knee OA, we included 1) KOOS pain scores (0–100), 2) cut-off of the KOOS pain score of ≤72 (KOOSpain ≤72) [13] and 3) a cut-off of ≤85 on at least 2 out of the 4 KOOS subscales pain, symptoms, ADL and QoL (modified Luyten) [28].

### 2.3 Statistics

All statistical calculations were carried out using SPSS version 29 (IBM corporation, New York, USA). Data were checked for normal distribution using visual inspection of histograms and Q-plots and interpretation of skewedness and kurtosis (±2). All data met the assumptions of normality except for the time between injury and reconstruction. Associated injuries were dichotomized where no associated injury was given the value 0, and the presence of any of the following additional injuries at the time of ACL injury was given the value of 1: meniscal, cartilage (International Cartilage Repair Society grade ≥3) and/or medial/lateral collateral ligament.

The Independent T-test, Chi^2^-test and Mann-Whitney *U* test were used as appropriate to assess differences in patient characteristics between those who responded and those who were lost to follow-up. Descriptive statistics [mean (SD), median (quartiles) and proportions] for patient characteristics and all outcomes, as well as the proportion of individuals meeting the criteria for early knee OA symptoms are reported.

Crude associations between patient characteristics and KOOS pain scores at 1 year and 3 years were calculated using Pearson's correlation coefficient (r) (continuous data), or Spearman's rank correlation coefficient (r_s_) (ordinal, non-normal distributed data). If more than one variable had a statistically significant association with KOOS pain scores, these variables were entered into a multivariable linear regression model (Enter method), with the specific participant characteristic variables as independent variables and KOOS pain score as dependent variable. To assess potential collinearity between the included independent factors, the variance inflation factor ​< ​4 was used.

Crude differences in patient characteristics between those meeting vs. those not meeting the criteria indicative of early knee OA symptoms at 1 year and 3 years post ACLR, respectively, were calculated using the independent T-test (continuous data), the Mann-Whitney *U* test (ordinal, non-normal distributed data) or Chi-square test (dichotomous data). No multivariable modelling was performed for the KOOSpain ≤72 criterion or modified Luyten criterion due to the low sample size for the group with early OA symptoms (KOOSpain ≤72) and the non-symptomatic group (modified Luyten), respectively.

Due to the few participants that had an ACLR using a quadriceps graft (n ​= ​7) or allograft (n ​= ​3), the analysis of associations between graft type and KOOS pain scores was limited to hamstring and patella grafts only. A 95 ​% confidence interval excluding zero or p-value ≤0.05 was considered statistically significant.

## 3 Results

### 3.1 Participant characteristics

Participant characteristics at 1-year post ACLR are reported in Table 1. Of the 106 participants included in this cohort study, 101 responded to the KOOS questionnaire at 1 year and eighty-six (81 ​%) responded at follow-up 3 years post ACLR. Of those, 11 (13 ​%) participants reported 13 serious reinjuries to the ipsilateral knee (graft rupture n ​= ​3, medial meniscus n ​= ​8, lateral meniscus n ​= ​1, caput fibula fracture n ​= ​1) and two participants reported an injury to the contralateral knee (ACL n ​= ​2) at follow-up. There were no statistically significant differences in 1-year characteristics between those who responded and those who were lost to follow-up (Table 2).

**Table 1:** Characteristics for the SHIELD cohort 1-year post ACLR.

1-year characteristic (baseline)	SHIELD cohort
Age *mean (SD)* (n ​= ​106)	25 (5)
Sex *females n (%)* (n ​= ​106)	53 (50)
BMI *mean (SD)* (n ​= ​104)	24.7 (3.9)
Time between injury and surgery (months) *median (quartiles)* (n ​= ​103)	7.5 (4–16.5)
Graft type *n (%)*	
*Hamstring*	67 (63)
*Patella*	29 (27)
*Quadriceps*	7 (7)
*Allograft*	3 (3)
Injury mechanism *non*-*contact n (%)* (n ​= ​102)	69 (68)
Associated injuries (any) *n (%)* (n ​= ​103)	75 (73)
*Cartilage (ICRS* ​≥ ​*3)*	16 (16)
*Medial meniscus*	48 (46)
*Lateral meniscus*	46 (44)
*MCL/LCL*	6 (6)
Family history of knee OA *n (%)* (n ​= ​105)	34 (32)
Pre-injury activity level *median (quartiles)* (n ​= ​101)	8 (5–9)
Current activity level *median (quartiles)* (n ​= ​101)	4 (3–7)
Returned to sport *n (%)* (n ​= ​95)	45 (47)

SD ​= ​standard deviation, BMI ​= ​Body Mass Index, OA ​= ​Osteoarthritis, ICRS ​= ​the International Cartilage Repair Society, MCL ​= ​medial collateral ligament, LCL ​= ​lateral collateral ligament.

**Table 2:** 1-Year characteristics for those who responded and those who did not or were lost to follow-up.

1-year characteristic (1 year post ACLR)	Responded (n ​= ​86)	Non-responders (n ​= ​20)^f^	p-value
Age *mean (SD)*	25 (5)	25 (5)	0.972^a^
Sex *females n (%)*	46 (53)	7 (35)	0.214^b^
BMI *mean (SD)*	24.7 (3.9)	24.9 (4.7)	0.844^a^
Time between injury and surgery (months) *median (quartiles)*	7.5 (4–14.5)	8.5 (2.5–32.25)	0.860^c^
Injury mechanism *contact n (%)*	26 (31)	7 (35)	0.518^b^
Graft type *n (%)*			
*Hamstring*	55 (65)	12 (57)	0.808^b^
*Patella*	22 (26)	7 (33)	
*Quadriceps*	6 (7)	1 (5)	
*Allograft*	2 (2)	1 (5)	
Associated injuries (any) *n (%)*	61 (73)	14 (74)	0.925^b^
Family history of knee OA *n (%)*	28 (33)	6 (32)	0.934^b^
Pre-injury activity level *median (quartiles)*	7 (5–9)	9 (7–9)	0.069^c^
Current activity level *median (quartiles)*	4 (3–7)	4 (2.25–6)	0.236^c^
Returned to sport (any level) *n* (%)	38 (48)^d^	7 (47)^e^	0.953^b^
1-year KOOS scores	n ​= ​85	n ​= ​16	
Subscale pain	83.3 (15.7)	82.4 (16.0)	0.823^a^
Subscale symptom	64.2 (16.2)	70.4 (15.2)	0.160^a^
Subscale ADL	92.5 (12.4)	91.1 (14.3)	0.697^a^
Subscale QoL	57.0 (20.6)	57.6 (19.7)	0.918^a^
Early knee OA (KOOS pain≤72) *n* (%)	15 (18)	3 (19)	0.932^b^
Early knee OA (at least 2 out of 4 KOOS subscales ≤85) *n* (%)	70 (82)	13 (81)	0.916^b^

SD ​= ​standard deviation, BMI ​= ​Body Mass Index, OA ​= ​Osteoarthritis, KOOS ​= ​Knee injury and Osteoarthritis Outcome Score, ADL ​= ​Activity of Daily Living, QoL ​= ​quality of life.

a Independent T-test.

b Chi^2^-test.

c Mann-Whitney *U* test.

d n ​= ​80.

e n ​= ​15 (not all responded to the return to sport question).

f The non-responder group includes 5 participants not responding to the questionnaire at both time-points (1- and 3-year).

### 3.2 Symptoms indicative of early knee OA

Mean (SD) KOOS pain scores were 83.2 (15.7) and 87.3 (12.7) at 1- and 3-year, respectively. Eighteen (18 ​%) and 14 (16 ​%) participants were defined as having symptoms of early knee OA by the KOOSpain ≤72 criterion at 1 year and 3 years post ACLR, respectively. When using the modified Luyten criterion, the corresponding proportions were n ​= ​83 (82 ​%) at 1 year post ACLR and n ​= ​67 (78 ​%) at 3 years post ACLR (Table 3). Seven of the 15 participants (47 ​%) and 59 of the 70 participants (84 ​%) that responded to the 3-year follow-up and were classified with early knee OA symptoms at 1 year were classified with early knee OA symptoms also at 3 years, using the KOOSpain ≤72 and modified Luyten criteria, respectively.

**Table 3:** KOOS subscales Mean (SD) values and proportions of participants with symptoms indicative of early knee OA at 1 year and 3 years post ACL reconstruction (ACLR).

	1-year (n ​= ​101)	3-years (n ​= ​86)
KOOS pain *Mean (SD) (%*≤*85)*	83.2 (15.7) (46)	87.3 (12.7) (33)
KOOS symptoms *Mean (SD) (%*≤*85)*	65.2 (16.1) (86)	66.9 (16.9) (84)
KOOS ADL *Mean (SD) (%*≤*85)*	92.3 (12.6) (20)	93.4 (11.0) (12)
KOOS QoL *Mean (SD) (%*≤*85)*	57.1 (20.4) (89)	66.3 (21.0) (85)
Early knee OA (KOOS pain≤72) *n* (%)	18 (18)	14 (16)
Early knee OA (at least 2 out of 4 KOOS subscales ≤85) *n* (%)	83 (82)	66 (77)

KOOS=Knee injury and Osteoarthritis Outcomes Score, OA ​= ​osteoarthritis, ACLR ​= ​anterior cruciate ligament injury, ADL ​= ​activity of Dayli living, QoL ​= ​quality of life.

### 3.3 Associations between participant characteristics and symptoms indicative of early knee OA

Higher age (r ​= ​−0.197, p ​= ​0.048), lower pre-injury (r_s_ ​= ​0.254, p ​= ​0.010) and 1-year activity level (r_s_ ​= ​0.348, p ​< ​0.001) were associated with worse KOOS pain scores at 1 year (Table 4) and were consequently entered into the regression model. The multivariable regression model suggested no associations between any of the patient characteristics and KOOS pain scores at 1 year when all variables in the model were considered. No collinearity between any of the independent variables was observed (VIF ≤1.48) (Table 5). Lower 1-year activity level was the only variable associated with worse KOOS pain scores at 3 years (r_s_ ​= ​0.239, p ​= ​0.028) (Table 5).

**Table 4:** Associations between baseline characteristics and KOOS pain scores at 1 year and 3 years post ACL reconstruction (ACLR).

1-year characteristic	KOOS pain scores 1 year post ACLR		KOOS pain scores 3 years post ACLR	
Age	r ​= ​−0.197, **p ​= ​0.048**, n ​= ​101		r ​= ​−0.189, p ​= ​0.081, n ​= ​86	
Sex mean (SD)	Females: 81.2 (16.0)	p ​= ​0.199^a^	Females: 86.6 (13.9)	p ​= ​0.567^a^
	Males: 85.3 (15.3)	n ​= ​101	Males: 88.2 (11.3)	n ​= ​86
BMI	r ​= ​−0.053, p ​= ​0.595, n ​= ​99		r ​= ​0.003, p ​= ​0.975, n ​= ​85	
Time between injury and surgery (months)	r_s_ ​= ​0.097, p ​= ​0.346, n ​= ​97		r_s_ ​= ​−0.049, p ​= ​0.658, n ​= ​84	
Injury mechanism *contact mean (SD)*	Contact: 83.8 (16.6)	p ​= ​0.903^a^	Contact: 87.7 (12.4)	0.938^a^
	Non-contact: 83.4 (14.6)	n ​= ​92	Non-contact: 87.9 (12.4)	n ​= ​79
Graft type *mean (SD)*	Hamstring: 81.3 (17.9)	p ​= ​0.148^a^	Hamstring: 86.2 (14.0)	p ​= ​0.343
	Patella: 85.7 (10.5)	n ​= ​101	Patella: 89.3 (10.8)	n ​= ​77
Associated injuries *mean (SD)*	Yes: 81.4 (17.6)	p ​= ​0.277^a^	Yes: 85.5 (16.1)	p ​= ​0.431
	No: 84.8 (13.5)	n ​= ​101	No: 88.2 (10.7)	n ​= ​86
Family history of knee OA *mean (SD)*	Yes: 81.4 (17.6)	p ​= ​0.277^a^	Yes: 85.5 (16.2)	p ​= ​0.364^a^
	No: 84.8 (13.5)	n ​= ​100	No: 88.2 (10.7)	n ​= ​86
Pre-injury activity level	r_s_ ​= ​0.254, **p ​= ​0.010**, n ​= ​101		r_s_ ​= ​0.181, p ​= ​0.098, n ​= ​85	
1-year activity level	r_s_ ​= ​0.348, **p**<**0.001**, n ​= ​101		r_s_ ​= ​0.239, **p ​= ​0.028**, n ​= ​85	
Returned to sport (any level) *mean (SD)*	Yes: 84.2 (16.7)	p ​= ​0.475^a^	Yes: 88 (12.6)	p ​= ​0.778^a^
	No: 81.9 (15.4)	n ​= ​95	No: 87.2 (12.2)	n ​= ​80

SD ​= ​standard deviation, KOOS=Knee injury and Osteoarthritis Outcomes Score, BMI=Body Mass Index, OA= Osteoarthritis, r ​= ​Pearson's correlation coefficient, r_s_ ​= ​Spearman's rank correlation coefficient, Bold characters indicate a statistically significant association (p ​≤ ​0.05).

a Independent T-test.

**Table 5:** Multivariable linear regression model for patient characteristics and KOOS pain at 1 year post ACLR.

Variables	KOOS pain at 1-year post ACLR (n ​= ​100)					
	B	SE B	95 ​% CI	β	p-value	R^2^ (adjusted R^2^)
Age	−0.35	0.32	−0.98–0.28	−0.12	0.271	0.084 (0.056)
Pre-injury activity level	0.03	0.83	−1.61–2.67	0.01	0.969	
1-year activity level	1.41	0.73	−0.04–2.86	0.18	0.057	

KOOS = Knee injury and Osteoarthritis Outcome Score, ACLR ​= ​anterior cruciate ligament reconstruction.

In univariable analysis, those presenting with symptoms indicative of early knee OA (KOOSpain ≤72) at 3 years post ACLR reported lower activity level at 1 year compared to those not presenting with early knee OA symptoms (median (quartiles) 3 (2–6) vs. 4 (3–7)) (Table 6). Those presenting with symptoms indicative of early knee OA at 1- and 3-years according to the modified Luyten criterion were more likely to have a hamstring graft than a patella graft (1 year: 75 ​% vs. 44 ​%, p ​= ​0.012, 3 year: 82 ​% vs 35 ​%, p ​< ​0.001) as well as reporting lower activity level at 1 year (median (quartiles) 1 year: 4 (3–7) vs. 5.5 (4–8), 3 year: 4 (3–6.5) vs. 7 (5–9)). Those presenting with symptoms indicative of early knee OA at 3-years were also approximately 3 years older than those who did not (mean diff (95 ​% CI) −2.9 (−5.6; −0.3) (Table 7). No other major differences in participant characteristics between those with and without symptoms indicative of early knee OA were observed at either 1 year or 3 years post ACLR (Table 6, Table 7).

**Table 6:** 1-Year characteristics for participants presenting with and without symptoms indicative of early knee OA 1 year and 3 years post ACL reconstruction (ACLR).

	Symptoms indicative of early knee OA 1 year post ACLR (KOOS pain≤72)			Symptoms indicative of early knee OA 3 years post ACLR (KOOS pain≤72)		
**1-year characteristic**	Yes (n ​= ​18)	No (n ​= ​83)	Mean diff (95 ​% CI)/p-value	Yes (n ​= ​14)	No (n ​= ​72)	Mean diff (95 ​% CI)/p-value
Age *mean (SD)*	27 (5.2)	25 (5.3)	−2.2 (−4.9; 0.56)^a^	27 (4.5)	25 (5.4)	−1.9 (−5.0; 1.1)^a^
Sex *n (%)*						
Females	12 (24)	39 (76)	p ​= ​0.142^b^	9 (20)	43 (80)	p ​= ​0.376^b^
Males	6 (12)	43 (88)		5 (13)	35 (87)	
BMI *mean (SD)*	24.0 (2.8)	24.7 (3.7)	0.7 (−1.1; 2.6)^a^	24.1 (3.3)	24.8 (3.8)	0.7 (−1.5; 2.8)^a^
Time between injury and surgery (months) *median (quartiles)*	7.5 (1.75–13.75)	8 (4–18.75)	p ​= ​0.616^c^	9 (5–11.5)	7 (4–16)	p ​= ​0.911^c^
Injury mechanism *n (%)* contact	6 (20)	24 (80)	p ​= ​0.906^b^	4 (15)	22 (85)	p ​= ​0.870^b^
Non-contact	10 (16)	51 (84)		8 (15)	45 (85)	
Graft type *n (%)*						
Hamstring	15 (23)	49 (77)	p ​= ​0.157^b^	10 (18)	45 (82)	p ​= ​0.747^b^
Patella	3 (11)	25 (89)		3 (14)	19 (86)	
Associated injuries *n (%)*						
Yes	14 (20)	56 (80)	p ​= ​0.221^b^	8 (13)	54 (87)	p ​= ​0.325^b^
No	2 (7)	25 (93)		5 (22)	18 (78)	
Family history of knee OA *n (%)*						
Yes	9 (27)	24 (73)	p ​= ​0.060^b^	6 (21)	22 (79)	p ​= ​0.369^b^
No	8 (12)	58 (88)		8 (14)	50 (86)	
Pre-injury activity level *median (quartiles)*	7 (3–8.25)	8 (6–9)	p ​= ​0.062^c^	7 (3.5–8)	8 (5–9)	p ​= ​0.106^c^
1-year activity level *median (quartiles)*	3 (1–6.25)	4 (3–7)	p ​= ​0.053^c^	3 (2–6)	4 (3–7)	**p ​= ​0.048**^c^
Returned to sport *n (%)*						
Yes	9 (21)	35 (79)	p ​= ​0.576^b^	4 (11)	34 (89)	p ​= ​0.286^b^
No	8 (16)	42 (84)		8 (19)	34 (81)	

SD ​= ​standard deviation, KOOS ​= ​Knee injury and Osteoarthritis Outcomes Score, BMI ​= ​Body Mass Index, OA ​= ​Osteoarthritis, Bold characters indicate a statistically significant difference.

a Independent T-test.

b Chi^2^-test.

c Mann-Whitney *U* test.

**Table 7:** 1-Year characteristics for participants presenting with and without symptoms indicative of early knee OA 1 year and 3 years post ACL reconstruction (ACLR).

	Symptoms indicative of early knee OA 1 year post ACLR (modified Luyten criterion)			Symptoms indicative of early knee OA 3 years post ACLR (modified Luyten criterion)		
**1-year characteristic**	Yes (n ​= ​83)	No (n ​= ​18)	Mean diff (95 ​% CI)/p-value	Yes (n ​= ​67)	No (n ​= ​19)	Mean diff (95 ​% CI)/p-value
Age *mean (SD)*	25.6 (5.2)	24.8 (5.6)	−0.8 (−3.5; 1.9)^a^	26.1 (5.1)	23.2 (5.1)	−2.9 **(-5.6; -0.3)**^a^
Sex *n (%)*						
Females	43 (52)	9 (50)	p ​= ​0.889^b^	36 (54)	10 (53)	p ​= ​0.932^b^
Males	40 (48)	9 (50)		31 (46)	9 (47)	
BMI *mean (SD)*	24.4 (3.4)	25.0 (4.3)	0.5 (−1.3; 2.4)^a^	24.6 (3.5)	24.8 (4.2)	0.2 (−1.7; 2.1)^a^
Time between injury and surgery (months) *median (quartiles)*	8 (4–16)	7 (4.75–18.25)	p ​= ​0.874^c^	9 (5–15)	6 (3–9)	p ​= ​0.074^c^
Injury mechanism *n (%)* contact	23 (30)	7 (44)	p ​= ​0.296^b^	20 (32)	6 (35)	p ​= ​0.813^b^
Non-contact	53 (70)	9 (56)		42 (68)	11 (65)	
Graft type *n (%)*						
Hamstring	58 (75)	7 (44)	**p ​= ​0.012**^b^	49 (82)	6 (35)	**p**<**0.001**^b^
Patella	19 (25)	9 (56)		11 (18)	11 (65)	
Associated injuries *n (%)*						
Yes	56 (70)	14 (78)	p ​= ​0.509^b^	46 (70)	16 (84)	p ​= ​0.210^b^
No	24 (30)	4 (22)		20 (30)	3 (16)	
Family history of knee OA *n (%)*						
Yes	29 (35)	4 (22)	p ​= ​0.283^b^	3 (34)	5 (26)	p ​= ​0.511^b^
No	53 (65)	14 (78)		44 (66)	14 (74)	
Pre-injury activity level *median (quartiles)*	7 (5–9)	9 (6.75–9)	p ​= ​0.268^c^	7 (4–9)	8 (7–9)	p ​= ​0.067^c^
1-year activity level *median (quartiles)*	4 (3–7)	5.5 (4–8)	**p ​= ​0.011**^c^	4 (3–6.25)	7 (5–9)	**p ​= ​0.002**^c^
Returned to sport *n (%)*						
Yes	34 (44)	11 (65)	p ​= ​0.114^b^	27 (44)	11 (58)	p ​= ​0.273^b^
No	44 (56)	6 (35)		35 (56)	8 (42)	

SD ​= ​standard deviation, KOOS ​= ​Knee injury and Osteoarthritis Outcomes Score, BMI ​= ​Body Mass Index, OA ​= ​Osteoarthritis, Bold characters indicate a statistically significant difference.

a Independent T-test.

b Chi^2^-test.

c Mann-Whitney *U* test.

## 4 Discussion

The result of this study showed that when using the KOOSpain ≤72 criterion, 18 ​% (1 year) and 16 ​% (3 years) of the participants were classified as having symptoms indicative of early OA after ACLR. The corresponding proportions for the modified Luyten criteria were 82 ​% (1 year) and 78 ​% (3 years). This indicates poor agreement between the different criteria. There was a low association between lower 1-year activity level and worse KOOS pain scores at 3 years, but no other statistically significant associations between any of the participant characteristics and KOOS pain at 1 year or 3 years post ACLR.

It is still debated how to define OA early in the disease process and in 2021, the Osteoarthritis Research Society International, thus, started an initiative for developing classification criteria for early-stage knee OA [29]. This initiative is, however, still in an early phase [3,4]. In the current study, we used two previously proposed criteria: The ≤72 on the KOOS pain subscale [13] and the modified Luyten criterion (≤85 on at least 2 out of the 4 KOOS subscales) [[14], [15], [16]] to classify participants with early OA symptoms 1 year and 3 years post ACLR. Our result showed major criteria dependent differences, where approximately 15 ​% were classified as having early knee OA symptoms when the KOOSpain ≤72 criterion was used, whereas the corresponding proportion was ∼80 ​% for the modified Luyten criterion at both time points. The 15 ​% is quite similar to the proportion of 9–10 ​% that Wasserstein et al. [13] and Ware et al. [8] reported using this criterion 2–7 years post ACLR. The proportion of individuals classified as having early knee OA using the modified Luyten criterion was, however, much higher in our cohort compared to previous studies that reported proportions of 36 ​% at 6 months [15] and 21 ​% 5 years [16] post ACLR. Arhos et al. [16] included only level 1 and 2 athletes 5 years post ACLR and excluded those with concomitant symptomatic meniscal as well as cartilage injuries. In the current study, we applied no criterion on activity level and included those both with and without associated injuries 1 year post ACLR, which may explain some of the variation between that cohort (21 ​%) and our cohort (80 ​%). In the study by Harkey et al., only 36 ​% of the participants met the modified Luyten criteria short-term (5–7 months) post ACLR [15]. Given that the KOOS scores presented in the current study are comparable to those reported in the Swedish National Knee Ligament Registry 1- to 2-years post ACLR (symptoms: 78, pain: 85, ADL: 92, function: 67, QoL: 62) [30], the explanation for the proportional differences compared to our study is unclear.

The differences in the classification of individuals are attributed to how these criteria were constructed. While the criterion proposed by Wasserstein et al. [13] is more conservative (cut-off ≤72) and only considers the pain subscale in the KOOS questionnaire, the Luyten criteria classifies early symptomatic OA as a score of ≤85 of *any* two of the four KOOS subscales pain, symptoms, ADL and QoL [14]. In the current study, the classification of early knee OA symptoms according to the modified Luyten criterion was driven by the symptoms and QoL subscales and not the KOOS pain subscale, where 89 ​% (symptoms) and 86 ​% (QoL) of the participants scored below the ≤85 cut-off. This may explain the differences in the classification of early knee OA symptoms between the modified Luyten criterion and the KOOSpain ≤72 criterion in this cohort.

Illingworth et al. [9], suggest that no single variable, such as KOOS pain, can be the primary sinner, but a combination of different variables is needed to get the full picture of early knee OA. Many experts still propose using pain as a clinical sign of knee OA and suggest that the KOOS score is the most appropriate tool [14] and KOOS pain has, indeed, been reported to be moderately related to structural changes such as cartilage loss [9]. However, it could be argued that using self-reported knee pain as a sole indicator for OA symptoms is not enough and needs to be supplemented by other clinical signs.

The original criteria proposed by Luyten et al., included a clinical examination with the presence of either joint line tenderness or crepitus as well as a Kellgren & Lawrence grade of ≤1 in addition to the KOOS criterion [14]. Since the prevalence of radiographic OA is reported to be between 6 ​% (1 year) and 21 ​% (5 year) post ACLR [31], it is likely that most of the participants in our study met also the radiographic criterion at 1 year but fewer at 3-years. However, by including more clinical measures, such as joint effusion and Heberden's nodes, in addition to joint line tenderness and crepitus, Mahmoudian et al. further improved the classification of participants with early knee OA using the Luyten criterion [28]. Although the KOOS symptoms scale includes a question regarding crepitus, the modified version of the Luyten criteria used in the current study does not require other clinical signs (e.g., joint line tenderness, Heberden's nodes) or Kellgren & Lawrence grade into account, thus leading to a high proportion of individuals classified as having early knee OA. Although symptoms and QoL are highly important outcomes, it is questionable if, without consideration of other clinical signs, a score of ≤85 on these subscales, should be interpreted as early OA 1–3 years post ACLR. The KOOS scores in the current study are comparable to scores reported up to five years post ACLR [30] whereas these scores seem to improve and be above 85 for all subscales except QoL at 10 years [32] limiting the interpretation of these scores as early OA. The ≤85 cut-off should also be seen in the light of the normative values for the KOOS QoL score (between 83 and 85) for the general population in this age group (18–34 years) [33]. These symptoms and impact on QoL may, thus, be associated with residual symptoms from the knee injury, subsequent surgery, the ability to perform previous sporting activities and possible changes in athletic identity and self-image which have previously been linked to knee-related QoL in this population [[34], [35], [36]].

Wasserstein et al. classified 9 ​% of the cohort as having early knee OA at both 2 years and 6 years post ACLR using the cut-off ≤72 on the KOOS pain subscale [13]. Although the proportions for our cohort were similar at 1 year and 3 years (18 ​% vs 14 ​%), on an individual level, we showed that more than half of the participants that were classified as having early knee OA symptoms at 1 year were no longer classified with early knee OA symptoms at 3 years (KOOSpain ≤72). When using the modified Luyten criterion, 16 ​% of the participants that were classified as having early knee OA symptoms at 1 year were not at 3 years. A previous study reported that 31 ​% of participants presented with features related to early knee OA on MRI (i.e., cartilage defects, osteophytes, bone marrow lesions) at 1 year post-ACLR [37]. While approximately half of the participants showed similar OA-related features at 5 years, 8 ​% (bone marrow lesions) to 31 ​% (cartilage defects) of the participants had a progression of these features, whereas 40 ​% of the bone marrow lesions had improved [31]. This indicates a role for MRI in detecting early OA in this population. Future studies will reveal if the criteria applied in this study are related to OA-related features on MRI. Given the mismatch of participants identified with the KOOSpain ≤72 criterion at the two time-points as well as between the two criteria, care should be taken when applying such criteria too soon after knee injury or knee surgery. Validation studies are warranted to investigate if any of these classification criteria at 1 year or 3 years after ACLR are associated with future OA-related structural changes.

Lower activity level at 1 year was associated with both more knee pain (KOOS pain scores) and being classified as having early knee OA symptoms (KOOSpain ≤72 and modified Luyten). This is in line with the study by Wasserstein et al. where an association between lower activity level at 2 years was associated with worse KOOS pain scores at 6 years post ACLR [13]. Previous research also found an association between early return-to-sport (<10 months) and future early knee OA features, such as bone marrow lesions [38]. Since there was no relation between return-to-sport status and KOOS pain scores in the current study, the explanation for the association between activity level and future knee pain is unclear. Further studies are needed to determine whether activity level per se predicts future OA symptoms or if those with more symptoms have a lower activity level. There was also an association between age and graft type and more symptoms according to the modified Luyten criterion where those classified as having early OA were slightly older and were more likely to have a hamstring graft than those who were not. This is contradictory to a previous meta-analysis reporting no difference in IKDC scores but less knee pain for those with a hamstring graft compared to patella graft at a minimum of 2 years post ACLR [39]. However, since we were not able to perform a multivariable analysis for this outcome, our results should be interpreted with caution. Given the few associations between patient characteristics and KOOS pain at either time point, future studies are warranted on the relation between other clinically relevant factors, such as e.g., muscle function and future symptoms indicative of early knee OA. Future studies with greater sample size may also consider comparing patient characteristics between those who were classified with early knee OA symptoms at neither time point with those who were classified with early knee OA symptoms at both time-points and with those who changed status over time.

The main strength of this study is that we included participants with different graft types, with and without associated injuries, and with different activity levels, constituting a clinically representative sample of patients. This study has some limitations. It is a descriptive study of patient characteristics from a longitudinal project investigating the relationship between muscle function and symptoms indicative of early knee OA after ACLR [24], and, as such, no a priory sample size calculations were performed for the research questions included in this study. While the sample size may be sufficient for investigating associations between patient characteristics and KOOS pain scores, it was not possible to perform multivariable modelling for the classification criteria for early knee OA symptoms (KOOSpain ≤72, modified Luyten) given the small number of participants classified as early OA symptoms, limiting the interpretation of this result. Almost half of those invited to participate in this study did not respond to the invitation. In addition, there was a drop-out rate of approximately 20 ​% at follow-up which may introduce selection bias to the results. The final cohort included in this study is, however, comparable with regards to sex, age, time from injury to surgery, and KOOS scores to the patients registered in the Swedish National ACL Register, which covers 90 ​% of all ACLRs performed in Sweden [30]. Furthermore, the drop-out analysis revealed no differences in patient characteristics or 1-year KOOS scores between those who responded and those who were non-responders, and, thus, we believe the participants in this study to be a representative sample of patients with ACLR.

## 5 Conclusion

The proportion of participants fulfilling suggested criteria for early knee OA symptoms after ACLR (16%–82 ​%) is highly dependent on the criteria applied and different criteria seem to capture varying aspects of symptoms. Given that more than half of the participants fulfilling the KOOSpain ≤72 criterion at 1 year did no longer fulfil this criterion at 3 years, caution is also needed when applying this criterion in this patient population. Lower physical activity level at 1 year may be associated with worse KOOS scores 3 years post ACLR but the small sample size limits interpretation of results.

## Author contributions

AC contributed to the conception and design of the study, collection of data, analysis and interpretation of data and was responsible for drafting the article. MAR, ME, and EA contributed to the conception and design of the study, interpretation of data and critical revision of manuscript drafts. DBS, PN and CJT contributed to data collection, interpretation of the data and critical revision of later draft versions. All authors approved the final version.

## Role of funding source

Funding for this study was provided by the 10.13039/501100007949Swedish Rheumatism Association, Governmental funding of clinical research within the National Health Services, 10.13039/100018740Anna-Greta Crafoord Foundation, the Greta and Johan Kocks foundation, and the Faculty of Medicine, Lund university.

## Declaration of competing interest

ME reports consultancy for Grunenthal Sweden AB and Key2Compliance AB. The other authors report no conflicts of interests relevant to the content of this manuscript.
